# Two-year long-term follow-up of treatment with the Optilume BPH catheter system in a randomized controlled trial for benign prostatic hyperplasia (The PINNACLE Study)

**DOI:** 10.1038/s41391-024-00833-z

**Published:** 2024-04-29

**Authors:** Steven A. Kaplan, Jared L. Moss, Sheldon J. Freedman

**Affiliations:** 1 Department of Urology, Icahn School of Medicine at Mount Sinai, New York City, NY USA; 2 Department of Urology, Ochsner-LSU Health Shreveport, Shreveport, LA USA; 3 Freedman Urology, Las Vegas, NV USA

**Keywords:** Prostatic diseases, Outcomes research

## Abstract

**Background:**

Patient outcomes were assessed 2 years after treatment with the Optilume BPH Catheter System, a minimally invasive surgical therapy for the treatment of lower urinary tract symptoms (LUTS) due to benign prostatic hyperplasia (BPH).

**Methods:**

One-hundred forty-eight adult males with symptomatic BPH were enrolled and randomized in a 2:1 fashion to Optilume BPH or Sham (100 Optilume BPH; 48 Sham). Long-term measures include International Prostate Symptom Score (IPSS), peak urinary flow rate (Qmax), Post-Void Residual Urine (PVR), quality of life measures and sexual function. Follow-up beyond one year was limited to those treated with Optilume BPH.

**Results:**

At 2 years, 67.5% (56/83 CI 56.3%, 77.4%) of participants in the Optilume BPH arm were symptomatic responders as defined by ≥30% improvement in IPSS without medical or surgical retreatment. IPSS significantly improved from 23.4 ± 5.5 (*n* = 100) to 11.0 ± 7.0 (*n* = 74). Qmax improved by 116.8.% (8.9 ± 2.2 (*n* = 97) to 19.0 ± 16.3 (*n* = 65)), while PVR showed a slight reduction (83.7 ± 70.3 (*n* = 99) to 65.9 ± 74.5 (*n* = 65)). Improvement in uroflowmetry measures was consistent across all prostate volumes. BPH-II improved from 7.0 ± 2.9 (*n* = 98) to 2.3 ± 2.5 at 1 year (*n* = 89) and remained consistent at 2.3 ± 2.9 at the 2-years (*n* = 74), representing a 53.9% improvement. IPSS QoL also improved from 4.6 ± 1.3 (*n* = 100) at baseline to 2.2 ± 1.5 (*n* = 74). The most common adverse events reported in the Optilume BPH arm were hematuria and urinary tract infection (UTI). No device and/or treatment related serious adverse events were reported occurring beyond 12 months post-procedure. There was no impact to sexual function.

**Conclusions:**

In the PINNACLE study, participants treated with the Optilume BPH Catheter System demonstrated continued and durable results at 2 years, affirming tolerability, safety, and the enduring effectiveness. The Optilume BPH Catheter System provides lasting results that are comparable to the more invasive therapies, while preserving the advantages with being a minimally invasive therapy.

**Registration:**

ClinicalTrials.gov NCT04131907.

## Introduction

Benign Prostatic Hyperplasia (BPH) along with accompanying lower urinary tract symptoms (LUTS) represents a common medical condition in aging males. LUTS is the most common reason for males to seek a urologist. As of 2015, the United States alone recorded 38.1 million cases of BPH and 21.3 million cases with an International Prostate Symptom Score (IPSS) exceeding 7. Of these, 12.2 million are under active management, predominantly through strategies such as watchful waiting (35%) and pharmaceutical interventions (54.8%) [1].

Treatment options for moderate to severe LUTS refractory to lifestyle modifications or medical management encompass surgical procedures or minimally invasive surgical therapies (MISTs). Surgical approaches interventions generally entail procedures such as open or laparoscopic prostatectomy, laser ablation, robotic water ablation, and transurethral resection of the prostate (TURP). Historically, TURP has been regarded as the benchmark for endoscopic BPH treatment, characterized by lower reoperation rates and notable enhancements in symptoms and urinary flow [2]. However, these resective treatments are invasive, typically necessitate inpatient hospitalization, and may not be suitable for individuals unable to tolerate anesthesia or potential procedure-related adverse effects.

Alternatively, there exist MISTs presently accessible for addressing BPH-related LUTS. These MISTs offer a less invasive approach for individuals afflicted by BPH who seek to preserve sexual function while obtaining relief from symptoms. MISTs that are accepted within clinical treatment guidelines for the treatment of BPH include transurethral incision of the prostate (TUIP), prostatic urethral lift (PUL), transurethral water vapor thermal therapy, and temporary implantable nitinol devices [3]. Results from randomized trials show that these therapies demonstrate similar improvements for International Prostate Symptom Score (IPSS) including quality of life (QoL) and peak urinary flow rate (Qmax) [4–7].

The Optilume BPH Catheter System (Urotronic, Inc./Laborie Medical Technologies, Plymouth, MN, USA) is an FDA-approved minimally invasive drug-coated balloon dilation system designed for addressing BPH-related LUTS. Its approval by the US Food and Drug Administration (FDA) in June 2023 marks a significant addition to the array of promising alternatives within the MIST category. Demonstrating comparable symptom improvement to other MISTs, the Optilume BPH Catheter System notably exhibited exceptional improvement in Qmax during a 1-year follow-up [8], surpassing outcomes observed with other MIST options [4–7].

Like other minimally invasive therapies such as PUL, transurethral water vapor therapy and implantable nitinol devices, treatment with the Optilume BPH Catheter System can be administered in an outpatient setting with multiple anesthesia options at the discretion of the treating healthcare provider. Other resective technologies like robotic water ablation therapy and TURP require general anesthesia in an operating room, typically includes an overnight hospital stay and may require invasive bleeding management such as a transfusion.

Optilume BPH is a safe and effective option, with excellent improvement in IPSS, quality of life measures (IPSS QoL and BPH-II) and Qmax through 1 year [8]. Here we present the results of the prospective multicenter, randomized clinical trial for patients treated with the Optilume BPH Catheter System that have completed 2-year follow-up.

## Materials and Methods

### Study Population

The PINNACLE study is a prospective, multicenter, randomized study evaluating the safety and effectiveness of the Optilume BPH Catheter System. The study was conducted at 18 investigation centers in North America (NCT04131907). One-hundred forty-eight adult males with symptomatic BPH were enrolled and randomized in a 2:1 fashion to Optilume BPH or Sham (100 Optilume BPH; 48 Sham). The research was conducted following the guidelines of the Declaration of Helsinki. Approval for the protocol was obtained from the institutional review boards at each participating site, and written informed consent was acquired from all participants before their involvement.

Eligibility criteria included individuals aged between 50 and 80 years with an International Prostate Symptom Score (IPSS) ≥ 13, a peak urinary flow rate (Qmax) between 5 and 12 mL/s, a prostate volume between 20 and 80 g, and a prostatic urethral length between 32 and 55 mm as determined by transrectal ultrasound (TRUS). Patients were excluded if they had undergone prior minimally invasive or surgical interventions on the prostate, had a prostate-specific antigen >10 ng/mL without a negative biopsy, had a diagnosis or suspicion of cancer in the prostate or bladder, or had a urinary tract diagnosis with potential impact on urinary function such as stricture. A period of washout of BPH medications (i.e., 6 months for 5-alpha reductase inhibitors (5-ARI) and 2 weeks for phosphodiesterase-5 (PDE5) inhibitors and alpha blockers was required prior to completion of baseline assessments. Prior to treatment, participants were counseled to abstain from sex or use barrier contraception for 30 days post treatment to avoid exposure of a sexual partner to paclitaxel.

### Treatment procedure

Briefly, the Optilume BPH catheter system consists of two dilation balloon catheters: one uncoated pre-dilation catheter and one drug-coated balloon catheter. During the Optilume BPH procedure, cystoscopy with a 20 F rigid scope is performed, followed by the insertion of the uncoated pre-dilation catheter under direct visualization. The correct placement of the catheter is aided by a blue mark visible on the catheter shaft, indicating its position at the distal end of the external sphincter. Subsequently, the pre-dilation catheter is dilated for approximately 1 min, creating the anterior commissurotomy. The drug-coated balloon is then inserted and placed similarly to the pre-dilation catheter, and it is inflated for at least 5 min. A Foley catheter is left in place for a minimum of 2 days after the completion of the procedure.

Participants were blinded to the treatment they received. Participants experiencing ongoing or recurrent LUTS secondary to BPH were counseled on treatment options by blinded site personnel. Participants who desired additional therapy had their blind broken to further discuss treatment options, including restarting medications, alternative MIST treatment, or TURP.

### Assessments and follow-up

After treatment, follow-up was required at 14 days, 30 days, 3 months, 6 months, and at 1 year after treatment in both arms. Participants randomized to the Sham group are followed to 1 year and discontinued from the study. Those randomized to the Optilume BPH treatment group are followed annually through 5 years. Assessments at annual follow-up include IPSS, BPH Impact Index (BPH-II), International Index of Erectile Function (IIEF), EQ-5D, Male Sexual Health Questionnaire (MSHQ) to assess ejaculatory function (EjD), and uroflowmetry to assess Qmax and post-void residual urine (PVR). A voided volume ≥150 mL was required to be considered a valid uroflowmetry assessment.

Participants in either group had the option to choose standard of care or other alternative treatments for persistent or recurring LUTS related to BPH at any point. Those in the Optilume BPH Catheter System group who decided to pursue alternative treatment were withdrawn from the study and monitored solely for general health outcomes for up to 5 years after treatment.

### Statistical analysis

Randomization was conducted using an electronic system employing permuted blocks stratified by center and baseline IPSS severity (≤19 vs. >19). Descriptive statistics were used to present study variables during follow-up, with continuous data presented as mean (SD) and categorical data as proportion (percent). Participants initially treated with Optilume BPH who require alternative therapy are considered treatment failures and are discontinued from the study. To accurately reflect the rate of symptomatic responders (≥30% improvement in IPSS and no medical or surgical retreatment), timepoints after study exit due to treatment failure are imputed as failures and included in the denominator. Confidence intervals for symptomatic responder rates are estimated using the Clopper-Pearson (exact) approach. P-values presented are nominal and not adjusted for multiple comparisons.

## Results

**Fig. 1:**
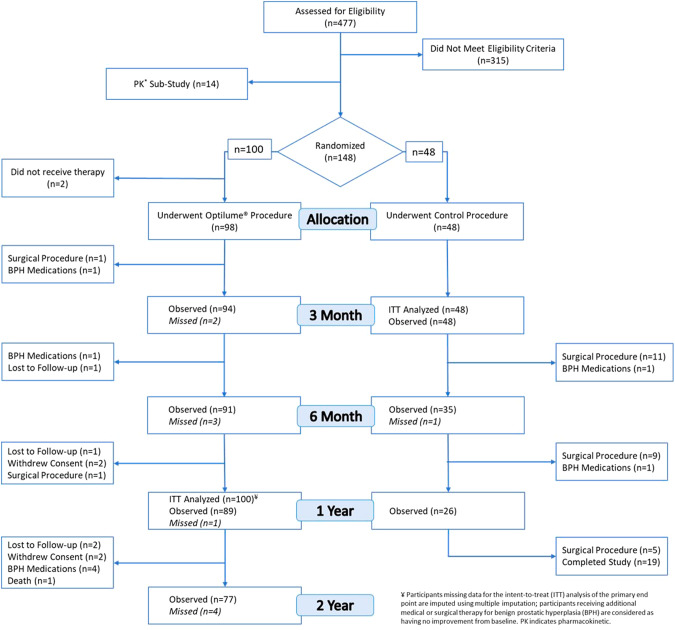
CONSORT (Consolidated Standards of Reporting Trials) flow diagram of participant disposition in both study arms through 2-year time point.

From February 2020 to September 2022, 148 participants were enrolled in the randomized cohort to receive Optilume BPH or Sham. The 48 Sham participants were followed for one year and then discontinued from the study unless they opted to cross over to treatment with Optilume BPH. Data for this group was previously summarized [8]. Participant demographics in both groups was well matched [8]. Participants in the Optilume BPH arm had an average age of 65.4 ± 6.4 years, average prostate volume of 44.9 ± 14.5 grams, average baseline IPSS of 23.4 ± 5.5. Baseline values for Qmax and PVR averaged 8.9 ± 2.2 mL/s and 84.1 ± 70.2 mL, respectively (Table 1). Of the 100 participants randomized to receive Optilume BPH, data for 77 participants are available for 2-year follow-up, and the results are presented here (Fig. 1). Of those 77 participants, 3 completed the two year follow-up visit, but IPSS was not done.

**Table 1:** Participant Demographics.

Characteristic	Optilume BPH
	(*n* = 100)
**Age (years)**	64.5 ± 6.4
**BMI (kg/m**^**2**^**)**	29.3 ± 4.5
**Prostate Specific Antigen (ng/mL)**	2.42 ± 2.0
**Prostate Volume (mL)**	44.9 ± 14.53
**Intravesical Prostatic Protrusion**	28.0% (28/100)
*IPP Size (mm)*	*5.1* ± *2.2*
**IPSS Score**	23.4 ± 5.5
**Qmax (mL/sec)**	8.9 ± 2.2
**Post-Void Residual Volume (mL)**	84.1 ± 70.2

### 2-year clinical outcomes

#### Efficacy

At 2 years, 67.5% (56/83 CI 56.3%, 77.4%) of participants who underwent treatment with Optilume BPH were symptomatic responders as defined by ≥30% improvement in IPSS without necessitating further medical or surgical retreatment. The denominator (*n* = 83) encompasses the IPSS data from participants currently enrolled in the study with a completed 2-year visit in addition to those who exited due to treatment failure. Among those with IPSS data observed at 2 years, 77% (57/74) of participants showed a symptomatic improvement characterized by ≥30% improvement in IPSS.

Participants who received treatment with the Optilume BPH Catheter System demonstrated a 50.8% improvement in IPSS from baseline to the 2-year follow-up. IPSS decreased from 23.4 ± 5.5 (*n* = 100) at baseline to 11.0 ± 7.0 (*n* = 74) at 2 years. Improvement was also observed in scores for both the storage (9.9 to 5.6, a decrease of 40.7%) and voiding (13.5 to 5.4, a decrease of 55%) domains of the IPSS. Of note, the proportion of participants with an IPSS < 8 at each follow-up interval, indicative of being mildly symptomatic, increased from 25% at 3 months (24/96), to 29.8% at 6 months (28/94), 30.9% at 1 year (29/94), and subsequently up to 32.5% at 2 years (27/83).

**Fig. 2:**
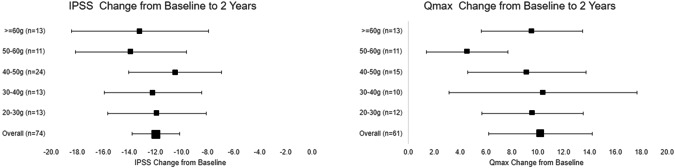
IPSS and Qmax Over Time by Prostate Size.

The uroflowmetry results, including Qmax and PVR, showed a notable improvement in the Optilume BPH group. Qmax improved by 116.8% from baseline (8.9 ± 2.2 (*n* = 97) to 19.0 ± 16.3 (*n* = 65)), while PVR showed a slight reduction from baseline (83.7 ± 70.3 (*n* = 99) to 65.9 ± 74.5 (*n* = 65)). Improvement in uroflowmetry measures was consistent across all prostate volumes (Fig. 2). Quality of life indicators in relation to urinary symptoms in the PINNACLE study include BPH-II score and IPSS-QoL. BPH-II improved from 7.0 ± 2.9 (*n* = 98) to 2.3 ± 2.5 (*n* = 89) at 1 year and remained consistent at 2.3 ± 2.9 (n = 74) at the 2-year follow-up, representing a 53.9% improvement (Table 2). IPSS QoL also improved from 4.6 ± 1.3 (*n* = 98) at baseline to 2.2 ± 1.5 (*n* = 74) at 2-year follow-up.

**Table 2:** Changes in Outcomes for Optilume BPH From Baseline Through 2 Years of Follow-up.

Measure	1 Month	3 Months	6 Months	12 Months	24 Months
**IPSS**					
No. (paired)	97	94	91	89	74
Mean ± SD (Baseline)	23.4 ± 5.5	23.3 ± 5.5	23.1 ± 5.5	23.0 ± 5.4	23.0 ± 5.6
Mean ± SD (Follow-up)	13.4 ± 7.0	12.6 ± 7.2	12.2 ± 7.1	10.9 ± 6.6	11.0 ± 7.0
Change ± SD	−10.0 ± 7.5	−10.7 ± 7.8	−10.9 ± 7.7	−12.1 ± 7.5	−12.1 ± 8.0
p-Value	<0.0001	<0.0001	<0.0001	<0.0001	<0.0001
**IPSS QoL**					
No. (paired)	97	94	91	89	74
Mean ± SD (Baseline)	4.6 ± 1.3	4.6 ± 1.3	4.6 ± 1.3	4.6 ± 1.3	4.6 ± 1.4
Mean ± SD (Follow-up)	3.0 ± 1.6	2.9 ± 1.7	2.6 ± 1.5	2.2 ± 1.5	2.2 ± 1.5
Change ± SD	−1.6 ± 1.9	−1.7 ± 1.8	−2.0 ± 1.8	−2.4 ± 1.9	−2.4 ± 1.9
p-Value	<0.0001	<0.0001	<0.0001	<0.0001	<0.0001
Qmax*					
No. (paired)	79	79	83	82	65
Mean ± SD (Baseline)	8.9 ± 2.2	8.8 ± 2.1	8.8 ± 2.2	8.7 ± 2.1	8.7 ± 2.1
Mean ± SD (Follow-up)	17.6 ± 9.0	18.8 ± 9.7	17.2 ± 8.9	19.0 ± 10.3	19.0 ± 16.3
Change ± SD	+8.7 ± 8.9	+10.0 ± 9.5	+8.3 ± 8.7	+10.3 ± 10.2	+10.3 ± 15.9
p-Value	<0.0001	<0.0001	<0.0001	<0.0001	<0.0001
PVR*					
No. (paired)	83	84	84	82	65
Mean ± SD (Baseline)	82.2 ± 72.1	84.8 ± 73.1	83.3 ± 72.8	83.2 ± 71.1	84.0 ± 72.6
Mean ± SD (Follow-up)	61.9 ± 55.5	66.8 ± 69.4	58.9 ± 59.3	58.0 ± 51.2	65.9 ± 74.5
Change ± SD	−22.5 ± 85.2	−19.2 ± 89.0	−25.7 ± 87.2	−25.2 ± 81.3	−18.1 ± 100.2
p-Value	0.019	0.053	0.009	0.006	0.151
**BPH-II**					
No. (paired)	96	93	91	89	74
Mean ± SD (Baseline)	6.9 ± 3.0	6.9 ± 3.0	6.8 ± 3.0	6.8 ± 3.0	7.0 ± 3.1
Mean ± SD (Follow-up)	5.3 ± 3.2	4.5 ± 3.2	2.9 ± 2.8	2.3 ± 2.5	2.3 ± 2.9
Change ± SD	−1.6 ± 3.8	−2.4 ± 3.8	−3.9 ± 3.8	−4.5 ± 3.2	−4.7 ± 3.7
p-Value	<0.0001	<0.0001	<0.0001	<0.0001	<0.0001

*Voided Volumes < 150 mL are excluded from analysis.

### Safety

#### Adverse Events through 2 years

The most common treatment related adverse events reported in the Optilume BPH treatment arm through 2-year follow-up were hematuria (39.8%, 39/98 participants) and urinary tract infection (UTI) (11.2%, 11/98 participants). A total of 41 hematuria events occurred in 39 subjects (39.8%) with most events being mild or moderate in severity (37/41, 90.2%) with a median time to resolution of 34 days. No transfusions were necessary for any of the instances of hematuria. The frequency of hematuria was reduced over time following the implementation of a hematuria management protocol which involved traction of the Foley catheter and flushing of the bladder after the procedure. No device and/or treatment related serious adverse events were reported occurring beyond 12 months post-procedure.

Overall Satisfaction (OS) scores for participants who are sexually active were collected as part of the IIEF and demonstrate no impact on sexual function. IIEF OS scores improved from 5.6 ± 2.7 (*n* = 96) at baseline to 6.5 ± 2.9 (*N* = 72) at 2 years. Scores from the erectile function domain of the IIEF also improved from 15.6 ± 10.3 to 20.4 ± 10.2 at 2 years (Table 3). Additionally, MSHQ-EjD scores in both ejaculatory function and ejaculatory bother were improved (Table 3).

**Table 3:** Sexual Function Parameters in Optilume BPH Subjects.

Measure		Group	Baseline	3 Months	6 Months	12 Months	24 Months
**IIEF**	Erectile Function	*n*	97	92	91	87	73
		Mean ± SD	15.6 ± 10.3	16.5 ± 10.8	19.3 ± 10.3	18.8 ± 10.7	20.4 ± 10.2
		Median	20	16.5	19	16	22
		Min, Max	1, 30	1, 30	1, 30	1, 30	1, 30
		Q1, Q3	6.0, 26.0	5.0, 28.0	5.0, 29.0	6.0, 29.0	8.5, 29.5
	Overall Satisfaction	*n*	96	92	91	86	72
		Mean ± SD	5.6 ± 2.7	6.2 ± 2.8	6.3 ± 2.9	6.3 ± 2.9	6.5 ± 2.9
		Median	6	6	6	6	7
		Min, Max	2, 10	2, 10	2, 10	2, 10	2, 10
		Q1, Q3	3.0, 8.0	4.0, 9.0	4.0, 10.0	4.0, 10.0	4.0, 9.0
**Measure**		**Group**	**Baseline**	**3 Month**	**6 Month**	**12 Month**	**2 Years**
**MSHQ**	Ejaculatory Function^a^	*n*	98	86	87	87	72
		Mean ± SD	7.5 ± 3.9	8.5 ± 4.8	8.3 ± 4.5	8.4 ± 4.6	8.6 ± 4.6
		Median	7	9	9	9	9
		Min, Max	1, 15	1, 15	1, 15	1, 15	1, 15
		Q1, Q3	5.0, 10.0	5.0, 13.0	5.0, 12.0	5.0, 12.0	4.5, 13.0
	Ejaculation Bother^b^	*n*	98	86	87	87	71
		Mean ± SD	2.5 ± 1.7	1.9 ± 1.6	2.1 ± 1.7	2.0 ± 1.7	1.8 ± 1.6
		Median	3	2	2	2	2
		Min, Max	0, 5	0, 5	0, 5	0, 5	0, 5
		Q1, Q3	1.0, 4.0	0.0, 3.0	0.0, 4.0	0.0, 3.0	0.0, 3.0

^a^Higher score = Less ejaculation dysfunction (Possible Range 1–15).

^b^Higher score = Greater bother with ejaculation difficulties (Possible Range 0–5).

## Discussion

PINNACLE is a prospective, multicenter, randomized study evaluating the safety and effectiveness of the Optilume BPH Catheter System. The 2-year results of the PINNACLE study confirm that treatment with Optilume BPH continues to be safe and has sustained long-term results that align with what was previously reported at 1 year [8]. 67.5% (56/83) of participants achieved success as symptomatic responders (i.e., ≥ 30% improvement in IPSS without further medical or surgical intervention). Surgical re-intervention occurred in 3% (3 out of 100) of subjects assigned to the Optilume BPH treatment arm (including prostatic artery embolization (PAE), TURP, and laser ablation). Introduction of pharmacotherapy post-Optilume BPH treatment was observed in 6% (6 out of 100) of participants, encompassing alpha blockers, PDE5 inhibitors, 5-ARIs, and supplements. Enhancements in other parameters including symptoms, quality of life measures, urine flow, are also shown to be maintained at 2 years.

Average IPSS significantly improved through 2 years (−12.0 ± 8.0). Qmax also improved by +10.3 mL/sec which exceeds what has been considered a clinically meaningful improvement of 2 mL/sec [9]. PVR was improved by 118.1 mL from baseline through 2 year follow-up.

The improvement of symptoms and urinary flow was observed across the full range of prostate volumes included in the study. Prostate volume ranged from 20.6 g to 76.1 g. (Fig. 2). While this may provide preliminary indication of the suitability of Optilume BPH for larger prostate volumes, further investigation is needed for prostate larger than 80 g. While the exclusion of prostate volumes >80 g may be considered a limitation of the PINNACLE study, it is also representative of the eligibility criteria for other minimally invasive therapies as many of those therapies are also not indicated for larger prostate volumes [10].

Quality of life measures were also positively impacted for participants in the study treated with the Optilume BPH Catheter System. IPSS-QoL and BPH-II both improved through 2 years. Sexual function was preserved as overall satisfaction scores of the IIEF improved from baseline. Erectile function was not adversely impacted. Improvement was observed in the sexual desire, intercourse satisfaction, and erectile function domain scores of the IIEF questionnaire. There were also no treatment-related serious adverse events reported.

The Optilume BPH Catheter System stands out due to its unique combination of minimally invasive treatment and notable enhancements in urinary flow, surpassing the effectiveness of other minimally invasive alternatives in this regard. This treatment is particularly suitable for individuals seeking to avoid or unable to tolerate general anesthesia. Furthermore, it presents an attractive option for patients as a therapy that does not involve resective or ablative tissue removal or implant placement. The device combines the procedural advantage of balloon dilation with a localized transfer of paclitaxel to limit hyperactive cell proliferation at the treatment site during the healing process. This dual action mode allows the Optilume BPH Catheter System to achieve the intended effect to open the narrowed urethra and maintain its patency.

Subsequent publications stemming from the PINNACLE study will provide ongoing updates on the long-term follow-up of individuals treated with the Optilume BPH Catheter System.

## Limitations

One limitation of the study is the exclusion criteria, which did not include patients with prostate volumes exceeding 80 g. Additionally, individuals with artificial urinary sphincters or urethral/prostatic stents, prior minimally invasive prostate interventions, a significant median lobe component (intravesical prostatic protrusion >1 cm), or a history of pelvic radiation were also excluded. The performance of the Optilume BPH Catheter System in these specific groups remains unknown, presenting an opportunity for further investigation in future studies.

## Conclusion

In the PINNACLE study, participants treated with the Optilume BPH Catheter System demonstrated strong improvement in symptoms and function outcomes, affirming both the procedure’s tolerability and the enduring effectiveness of the outcomes. Subjects treated with the Optilume BPH Catheter System achieved the most significant reported enhancement in peak urinary flow rate (Qmax) among clinical trials evaluating minimally invasive devices for BPH. Furthermore, it exhibited a safety profile comparable to other minimally invasive therapies [4–8]. The Optilume BPH Catheter System provides lasting 2-year results that are comparable to the more invasive therapy options, while preserving the advantages with being a minimally invasive therapy.

## Data Availability

The datasets generated and/or analysed during the current study are available from the corresponding author on reasonable request.

## References

[1] Vuichoud C, Loughlin KR. Benign prostatic hyperplasia: epidemiology, economics and evaluation. Can J Urol [Internet]. 2015;22:1–6. 26497338

[2] He W, Ding T, Niu Z, Hao C, Li C, Xu Z, et al. Reoperation after surgical treatment for benign prostatic hyperplasia: a systematic review. Front Endocrinol [Internet]. 2023;14:1287212. 10.3389/fendo.2023.1287212 PMC10665564 38027158

[3] Lerner LB, McVary KT, Barry MJ, Bixler B, Dahm P, Das AK, et al. Management of lower urinary tract symptoms attributed to benign prostatic hyperplasia: AUA GUIDELINE PART I, initial work-up and medical management. J Urol. 2021;206:806–17. 34384237 10.1097/JU.0000000000002183

[4] Roehrborn CG, Gange SN, Shore ND, Giddens JL, Bolton DM, Cowan BE, et al. The prostatic urethral lift for the treatment of lower urinary tract symptoms associated with prostate enlargement due to benign prostatic hyperplasia: the L.I.F.T. Study. J Urol [Internet]. 2013;190:2161–7. 23764081 10.1016/j.juro.2013.05.116

[5] McVary KT, Gange SN, Gittelman MC, Goldberg KA, Patel K, Shore ND, et al. Minimally invasive prostate convective water vapor energy ablation: a multicenter, randomized, controlled study for the treatment of lower urinary tract symptoms secondary to benign prostatic hyperplasia. J Urol [Internet]. 2016;195:1529–38. 26614889 10.1016/j.juro.2015.10.181

[6] Chughtai B, Elterman D, Shore N, Gittleman M, Motola J, Pike S, et al. The iTind temporarily implanted nitinol device for the treatment of lower urinary tract symptoms secondary to benign prostatic hyperplasia: a multicenter, randomized, controlled trial. Urol [Internet]. 2021;153:270–6. 33373708 10.1016/j.urology.2020.12.022

[7] Pisco JM, Bilhim T, Costa NV, Torres D, Pisco J, Pinheiro LC, et al. Randomised Clinical Trial of Prostatic Artery Embolisation Versus a Sham Procedure for Benign Prostatic Hyperplasia. European Urology [Internet]. 2019 Dec [cited 2020 Jan 15]. 10.1016/j.eururo.2019.11.010 31831295

[8] Kaplan SA, Moss J, Freedman S, Coutinho K, Wu N, Efros M, et al. The PINNACLE Study: a double-blind, randomized, sham-controlled study evaluating the optilume BPH catheter system for the treatment of lower urinary tract symptoms secondary to benign prostatic hyperplasia. J Urol. 2023;210:500–9. 37555604 10.1097/JU.0000000000003568 PMC12721621

[9] National Institute for Health and Care Excellence. Overview | Lower urinary tract symptoms in men: management | Guidance | NICE [Internet]. Nice.org.uk. NICE; 2015. 31999413

[10] Foster HE, Barry MJ, Dahm P, Gandhi MC, Kaplan SA, Kohler TS, et al. Surgical management of lower urinary tract symptoms attributed to benign prostatic hyperplasia: AUA guideline. J Urol [Internet]. 2018;200:612–9. 29775639 10.1016/j.juro.2018.05.048

